# The theory of event coding (TEC) as embodied-cognition framework

**DOI:** 10.3389/fpsyg.2015.01318

**Published:** 2015-09-01

**Authors:** Bernhard Hommel

**Affiliations:** Cognitive Psychology Unit, Institute of Psychology, Leiden Institute for Brain and Cognition, Leiden UniversityLeiden, Netherlands

**Keywords:** embodied cognition theory, cognitivism, perception and action, distributed cognition, human cognition, theory, perception for action

## Abstract

The concept of embodied cognition attracts enormous interest but neither is the concept particularly well-defined nor is the related research guided by systematic theorizing. To improve this situation the theory of event coding (TEC) is suggested as a suitable theoretical framework for theorizing about cognitive embodiment—which, however, presupposes giving up the anti-cognitivistic attitude inherent in many embodiment approaches. The article discusses the embodiment-related potential of TEC, and the way and degree to which it addresses Wilson’s (2002) six meanings of the embodiment concept. In particular, it is discussed how TEC considers human cognition to be situated, distributed, and body-based, how it deals with time pressure, how it delegates work to the environment, and in which sense it subserves action.

The general intuition that human cognition is somehow “embodied” is widely shared and has stimulated various new brands of research. And yet, the concept of embodied cognition is ill-defined and a general, testable theory of its underlying mechanisms has not yet been presented. This has rendered tests of the concept difficult and often metaphorical in nature, which stands in the way of broader acceptance. The main reason for that, so I claim, is the anti-cognitivistic attitude of most embodiment approaches. Not only is this attitude more rooted in ideology than in empirical data, but it also prevents embodiment approaches from using cognitivistic tools to build mechanistic theories that provide the badly needed testable hypotheses. In the following, I shall argue that some cognitivistic approaches are well-equipped to capture the essence of cognitive embodiment. In particular, I shall argue that the theory of event coding (TEC; Hommel et al., 2001a) provides almost all that embodiment theories need, and that it therefore provides a mechanistic approach to the embodied-cognition concept—which TEC fully embraces. I shall demonstrate that by going through the six different meanings of embodied cognition that Wilson (2002) has identified in the literature, and explain how TEC fits with (the unideological aspects of) these six meanings.

## The Theory of Event Coding

**FIGURE 1:**
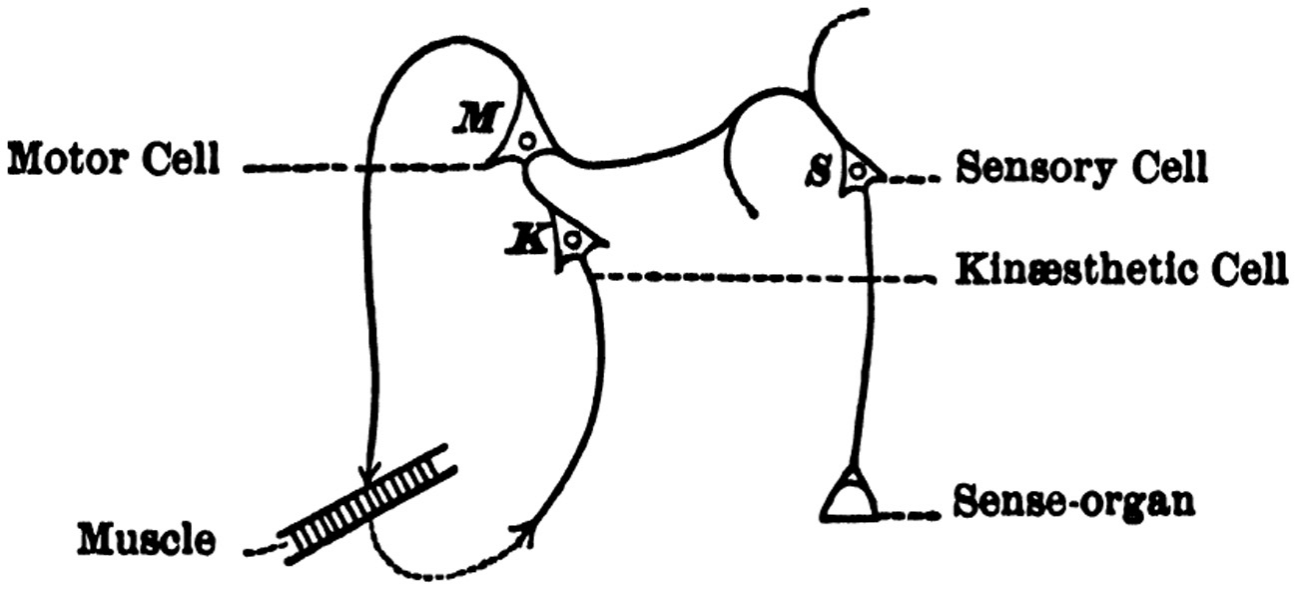
**James’ (1890, p. 582) neural model of acquiring ideomotor control.** Random activation of motor units (M) leads to non-voluntary motor activity (motor babbling), which in turn produces external effects (changes in body, environment, and body-environment relationship) that are registered by sensitive receptors (e.g., K—a kinesthetic receptor). Frequent co-activation of motor patterns (M) and receptors picking up the thereby produced re-afferent information (K) will lead to bidirectional associations between the two (M↔K). As a consequence, the motor pattern can be controlled/activated by endogenously re-activating the sensory part—i.e., by just “thinking of” or imagining a wanted sensory action effect. That is, internal reactivation of K (or codes of another sensory modality) serves to control M.

Theory of event coding is rooted in the cognitivistic ideomotor approaches of Lotze (1852), Harless (1861), and James (1890), and yet embraces the idea that human cognition emerges from sensorimotor processing. In contrast to behavioristic or information-processing approaches, ideomotor theory considers humans as active agents that perform actions to reach particular goals. Accordingly, the theoretical analysis does not start with stimuli but with goals (intended action effects), which are assumed to trigger the execution of movements suited to reach them. Goals are acquired by actively exploring the environment, which creates associations between motor activities and representations of their perceptual consequences (Elsner and Hommel, 2001). These action-effect bindings provide the basis for voluntary action: the agent only needs to “think of” the representation of a wanted action effect to activate the motor pattern needed to produce it (for overviews, see Hommel, 2009; Shin et al., 2010)—see **Figure 1**. Indeed, planning to produce particular action outcomes (e.g., hand movements or facial expressions) activates the neural codes of these consequences (areas EBR and FFA, respectively) before execution begins (Kühn et al., 2011).

Theory of event coding combines the ideomotor mechanism with assumptions about how perceptual and action events are represented. In particular, TEC comprises of four basic assumptions (Hommel et al., 2001a): (I) Perceptual events and planned actions are cognitively represented by *event codes*; (II) which are integrated assemblies of *feature codes*; (III) which in turn are cognitive/brain states correlated with external (perceived or self-generated) features (*distal coding*); (IV) so that the basic units of both perception and action (assemblies of feature codes) are *sensorimotor* entities, in the sense that they are activated by sensory input (=perception) and controlling motor output (=action).

What does it mean, from a TEC point of view, that cognition is embodied? As mentioned already, this depends on which sense of cognitive embodiment one refers to. According to Wilson (2002), at least six different meanings can be distinguished. In the following, I shall briefly introduce each of these criteria, carve out their less ideological essence, and show how they are met by TEC. For a broader, more detailed discussion, see Hommel (2015).

## Situated Cognition

The idea that cognitive activity is always situated and taking place in a particular context comes in two different flavors (Wilson, 2002). One relates to the philosophical/pedagogical claim that knowing is inseparable from doing, which is why education should favor learning-by-doing over the passive accumulation of knowledge (e.g., Greeno, 1998). This approach emphasizes the role of active agency in knowledge acquisition and internal representation—exactly as proposed by ideomotor theory and TEC. The ideomotor approach does not merely presume the existence of action goals or particular knowledge to account for voluntary action, but explains how goals are acquired through concrete hands-on exploration of one’s own body and its interactions with the environment (Verschoor et al., 2010). TEC extends this mechanism to a general theory of human cognition and claims that humans do not just acquire action goals through sensorimotor experience but that most or all knowledge is rooted in sensorimotor experience. It therefore seems to fit perfectly with the philosophical/pedagogical embodiment concept.

The other flavor of situated cognition comes from cognitive robotics (e.g., Clancey, 1997) and claims that agents may often not require internal information but can simply pick it up from their environment—which obviously borrows from Gibsonian Ecological Psychology and the assumption that environments provide action-related affordances for the active perceiver (Gibson, 1979). On the one hand, it is uncontroversial that humans and other primates possess (dorsal) online information-processing channels that feed more or less directly into action systems (Milner and Goodale, 2006), which may be considered a system that processes affordances (Michaels, 2000). On the other hand, however, there is no evidence that voluntary actions can be planned and carried out successfully without the help of other (ventral) off-line channels devoted to object identification, planning, and action evaluation—processes that rely on memory, which even in the case of procedural knowledge can be considered to represent past experiences.

It is this second off-line system responsible for setting up and planning voluntary actions that TEC is concerned with, while the theory has little to say about the subserving online channels (Hommel et al., 2001b). However, almost all of our everyday actions rely on previously acquired knowledge about how to use the available tools to reach our goals—just think of using a computer or coffee machine, or engaging in verbal communication and socially appropriate behavior. And they are often planned ahead in the absence of situational cues—which falls outside of the scope of (neo-) classical affordance-based approaches. As many actions require, or benefit from, the integration of knowledge-dependent off-line processes, and environmentally driven online processes, they can be considered goal-directed and context-sensitive at the same time. Accordingly, it makes sense to try integrating cognitivistic ideomotor theory and affordance-based approaches (Glover, 2004) rather than putting them into opposition—as some situated-cognition approaches tend to do (e.g., Pfeifer and Bongard, 2006).

## Cognition under Time Pressure

This brand of the embodied-cognition concept assumes that engaging in cognitive activities is particularly time costly and therefore unlikely to be the basis of everyday action. In cognitive robotics, this idea has been taken to suggest dropping the cognitive overhead so to allow robots to meet real-time constraints (e.g., Pfeifer and Scheier, 1999), and reasoning theorists (e.g., Gigerenzer et al., 1999) have used it to argue that people prefer cognitive shortcuts over full-fledged cognitive analyses of a problem.

One can argue whether time pressure is a real problem in humans: not only are there few everyday situations that would not allow taking the time for fuller cognitive analysis, but nature has also equipped us with reflexes that allow engaging in fight or flight long before grasping the actual situational demands. Hence, survival seems possible with the human mix of slow cognition and fast reflexes.

But what is more, theoretical analysis and empirical evidence suggest that slow cognition does not intervene between perception and action even if time allows (Hommel, 2013). Indeed, the main purpose of cognition seems to consist in anticipatory (oﬄine) preparation: selecting a goal, configuring the system for a particular task, priming goal-relevant action systems, and preparing for the processing of possible trigger stimuli. It is these preparatory processes that TEC is targeting. Once an action has been selected and sufficiently prepared, not much cognitive activity seems to go on and environmental information will commonly suffice to drive the action to completion—a kind of prepared reflex (Hommel, 2000). If, thus, cognition is not for online control but for off-line preparation, the possible slowness of cognitive processes does not serve as an argument against cognitivistic approaches and not even against (properly programmed) cognitive robots.

## Oﬄoading Cognitive Work onto the Environment

This blend of the embodiment approach tries to reduce the amount of knowledge that agents need to process. It assumes that the environment can serve as its own memory (e.g., Brooks, 1991; O’Regan and Noe, 2001), so that agents do not need to develop internal world models. Interestingly, supporting evidence comes exclusively from spatial tasks (Wilson, 2002), and it certainly makes sense that spatial decisions consider available spatial information. It is less clear how oﬄoading might work with actions like talking, dancing, or writing an article, but this may be the reason why the available evidence is restricted to less knowledge-heavy spatial actions. In any case, the model-less embodiment approach is fully consistent with TEC, which does not assume that actions rely on world models. While TEC aims to explain how people are planning goal-directed actions (based on procedural, implicit knowledge gathered through active experience), it does not claim that all aspects of actions are predetermined by planning. It rather assumes that planning is restricted to the specification of goal-relevant action outcomes (e.g., of the cup to be grasped for drinking), while the specification of goal-unrelated action features (e.g., the particular kinematics) is left to environmentally driven online channels that continuously feed in information during action execution (Hommel, 2010).

## Distributed Cognition

The claim that human cognition is not restricted to an individual’s mind and brain but involves the environment as well (e.g., Wilson and Golonka, 2013) can be considered another revival of a theme with a much older history (e.g., interactionism in personality psychology). According to Wilson (2002) the claim actually consists of two parts: that including the environment in analyses of human cognition provides more information than excluding it—which is true but too trivial to be controversial—and that excluding the environment from the analysis does not allow for any interesting insight into human cognition in principle.

It must be said that the distributed-cognition criterion has not yet been supported by any specific evidence. Neither has it been defined which aspects of the environment actually count (e.g., if a symbol on the monitor is enough, almost all cognitive research does take the environment into account), nor has it been demonstrated that, and in which sense the available cognitive/neurocognitive research has failed to produce meaningful results, nor has the approach itself produced any specific evidence to its own support—all the evidence that proponents tend to discuss was motivated by other than the distributed-cognition approach (e.g., see Wilson and Golonka, 2013). Hence, even the few observations in the literature that distributed-cognition proponents do find relevant did not require the distributed-cognition approach to make them.

However, a more liberal interpretation of the approach might get close to the situated-cognition criterion, and thus rightly attract attention to the relevance of concrete sensorimotor experience of the agent with his or her environment. This approach would make TEC and its reliance on sensorimotor experience a valuable tool to go beyond abstract complaints, and allow for the empirical test of concrete hypotheses about concrete phenomena.

## Cognition Subserves Action

The claim that human cognition evolved to subserve action has considerable Darwinistic face value: evolution operates on actions, not on ideas. It is therefore not surprising that cognition-for-action has been a dominant theme in many approaches, including American pragmatism, behaviorism, Russian activity theory, the motor theory of speech, and the mirror neuron approach. The concept also represents the very core of TEC, which partly reflects its intellectual heritage. And yet, the architectural and process-related assumptions underlying TEC make it unique in the field in a number of ways.

Theory of event coding shares the idea of cognition-for-action with respect to phylogenetic and ontogenetic considerations: the neural/functional architecture underlying the distribution of labor between dorsal and ventral information-processing streams is likely to reflect the importance of action in evolution and, as explained already, ontogenetic cognitive development relies on sensorimotor experience. However, TEC differs from other approaches in denying that every single use of cognitive skill or content must be accompanied by sensorimotor activity—as embodied-cognition proponents like Gallese and Goldman (1998) or Barsalou (2008) suggest (which is not to deny that these approaches share many other aspects with TEC).

One reason why cognition without sensorimotor activity or mental simulation should be possible relates to TECs intentional-weighting principle (Memelink and Hommel, 2013). TEC assumes that perceived or to-be-produced events are represented by means of event files—integrated bindings of the codes of distal event features (Hommel, 2004). The contribution of each component of such a binding is assumed to be weighted according to its situational relevance, so that codes related to the ringing sound of a telephone will be weighted more strongly than codes related to its color when waiting for a call. This means that event representations are tailored to the goal and task at hand, which implies that not all components of the representation are sufficiently activated to contribute to cognition and action. This means that, even though most cognitive representations are likely to comprise of both perceptual and action components, cognitive operations using these representations are possible without above-threshold activation of some components. If, thus, the cognitive operations do not require overt action (e.g., when processing words for silent reading), it is possible that they are carried out without measurable motor activity. In other words, sensorimotor activity is important for creating cognitive representations but not necessarily for using them.

The other reason is that grounding basic cognitive units in sensorimotor experience, as TEC assumes, does not necessarily prevent the creation of other representations that refer to combinations of, or relations between, such basic units. For instance, there is no reason to exclude that people are able to combine the (sensorimotorically grounded) representation of a horse with the (sensorimotorically grounded) representation of a horn to create the representation of a unicorn without ever having sensorimotorically experienced one. According to TEC, the resulting representations may be considered abstract but not necessarily symbolic or arbitrary, and they still can be considered as being grounded in sensorimotor experience.

## Body-Based Cognition for Off-Line Use

The claim refers to the idea that cognitive structures or skills that emerged through sensorimotor interactions with the environment could be used off-line—in the absence of overt behavior—to subserve cognitive activities (e.g., Glenberg, 1997). This claim also has a rather long history, especially in Russian activity theory (e.g., Vygotsky, 1962) and approaches that conceive of cognition as interiorized action. TEC fails to provide a systematic scenario of how interiorization might work in detail (and there is in fact no such theory available), but it does provide the necessary cognitive infrastructure. As explained already, TEC assumes that overt sensorimotor action leads to the binding of motor patterns and codes representing their consequences. Acquiring these bindings allows the agent to run them internally to simulate the action without actually activating the motor patterns (if intentional weighting deactivates the motor components). The acquisition of multiple sensorimotor events allows the agent to construct more complex event sequences, such as for making coffee (Kachergis et al., 2014). These representations provide information about how to move from one situation to another to reach a distant goal, which can be used to simulate and to compare alternative problem-solving strategies.

## Conclusion

Many embodied-cognition approaches have been put forward with a strong anti-cognitivistic attitude that they share with, and in some cases borrow from, behaviorism, ecological psychology, and evolutionary psychology, and indeed many of the ecological, and evolutionary arguments have resurfaced in the embodied-cognition debate (e.g., see Wilson and Golonka, 2013). This is unfortunate for two reasons. For one, most of these arguments are simply misdirected: they mainly challenge the symbol-heavy good old-fashioned artificial intelligence, which, however, had only negligible impact on modern cognitive psychology/neuroscience. And, for another, rejecting cognitivism prevents embodied-cognition theorists to develop mechanistic and therefore testable models that allow them to explore how fruitful the embodiment approach actually is. Without such models, no direct comparison with non-embodied approaches is possible, and thus no competition for the better explanation on cognitive psychology’s to-do list. Cherry-picking examples that minimize cognitive processes, knowledge, and internal preparation may help to illustrate basic principles, but scientific approaches to human cognition that are unable to account for everyday actions like making a phone call, preparing coffee, or asking for directions are unlikely to succeed.

I propose that a more constructive approach to embodied cognition is possible and probably more successful. As I have argued, a cognitivistic approach to the relationship between perception and action is likely to be useful for that purpose. In particular, the TEC is theoretically commensurable with all six of Wilson’s (2002) meanings of the embodiment concept. TEC assumes the existence of internal representations and claims that such representations are involved in producing actions, which makes it a cognitivistic approach. At the same time, it does not only assume that human cognition is situated, distributed, and body-based, but also explains how; it also explains how cognition deals with time pressure, how it is delegated to the environment, and which sense it subserves action. Extensions of the theory are possible and wanted on the way to a truly comprehensive framework of human cognition, and combining TEC with mechanistic models of online/affordance-based control and with assumptions about interiorization seems particularly promising in this respect.

## Conflict of Interest Statement

The author declares that the research was conducted in the absence of any commercial or financial relationships that could be construed as a potential conflict of interest.
